# Tumorigenic and Differentiation Potentials of Embryonic Stem Cells Depend on TGF*β* Family Signaling: Lessons from Teratocarcinoma Cells Stimulated to Differentiate with Retinoic Acid

**DOI:** 10.1155/2017/7284872

**Published:** 2017-07-16

**Authors:** Olga Gordeeva, Sergey Khaydukov

**Affiliations:** ^1^Kol'tsov Institute of Developmental Biology, Russian Academy of Sciences, 26 Vavilov Street, Moscow 119334, Russia; ^2^Shemyakin and Ovchinnikov Institute of Bioorganic Chemistry, Russian Academy of Sciences, Miklukho-Maklaya Street 16/10, Moscow 117997, Russia

## Abstract

A significant challenge for the development of safe pluripotent stem cell-based therapies is the incomplete in vitro differentiation of the pluripotent stem cells and the presence of residual undifferentiated cells initiating teratoma development after transplantation in recipients. To understand the mechanisms of incomplete differentiation, a comparative study of retinoic acid-induced differentiation of mouse embryonic stem (ES) and teratocarcinoma (EC) cells was conducted. The present study identified differences in proliferative activity, differentiation, and tumorigenic potentials between ES and EC cells. Higher expression of Nanog and Mvh, as well as Activin A and BMP4, was found in undifferentiated ES cells than in EC cells. However, the expression levels of Activin A and BMP4 increased more sharply in the EC cells during retinoic acid-induced differentiation. Stimulation of the Activin/Nodal and BMP signaling cascades and inhibition of the MEK/ERK and PI3K/Act signaling pathways resulted in a significant decrease in the number of Oct4-expressing ES cells and a loss of tumorigenicity, similar to retinoic acid-stimulated EC cells. Thus, this study demonstrates that a differentiation strategy that modulates prodifferentiation and antiproliferative signaling in ES cells may be effective for eliminating tumorigenic cells and may represent a valuable tool for the development of safe stem cell therapeutics.

## 1. Introduction

The cell derivatives of pluripotent stem cells are considered to be promising cell sources for regenerative therapy. Pluripotent stem cells of different origins are capable of unlimited self-renewal and differentiation into all types of somatic and germ cells in vitro and in vivo [1–9]. However, complete implementation of pluripotent potential is only possible when pluripotent stem cells are reintegrated with the blastocyst [6, 10–13]. In contrast, in vitro differentiation of the pluripotent stem cells is asynchronous and incomplete; and therefore, the residual undifferentiated cells can initiate teratoma development after transplantation into the tissues of an adult animal recipient [14–21]. This feature of pluripotent stem cells is one of the main issues for the development of safe pluripotent stem cell-based therapy.

Paradoxically, pluripotent embryonic stem (ES) and embryonic germ (EG) cells are the only types of genetically normal and nontransformed cells that can form tumors after transplantation into adult animal recipients. It is believed that genetically normal pluripotent stem cells form benign tumors that do not contain undifferentiated cells, whereas pluripotent stem cells carrying genetic aberrations give rise to malignant tumors with undifferentiated cells, similar to spontaneous teratocarcinoma tumors [22–24]. However, these correlations were found only for some human ES cell lines with abnormal karyotypes, whereas mouse ES cells did not show a strong correlation of their karyotypes or other genetic modifications (excluding transgenic mice with E-ras overexpression) and increased tumorigenicity and malignancy [25–27]. At the same time, mouse and human teratocarcinoma (EC) cells with different genetic disorders derived from spontaneous tumors are indeed capable of forming secondary malignancies after serial transplantation into recipients [28–34]. It can be assumed that the high risk of cancer initiation after transplantation of pluripotent stem cell-derived cells can be associated with mutations in oncogenes and tumor suppressor genes. Moreover, numerous studies have shown that long-term in vitro cultivation leads to the accumulation of genetic aberrations and abnormal epigenetic changes in the genome of pluripotent stem cells, including mutations in oncogenes and tumor suppressors [27, 35–40]. This property of in vitro-maintained cells is the second major problem delaying the clinical application of cellular technologies based on pluripotent stem cells.

Thus, to assess the risks and benefits of cellular technologies for regenerative medicine, it is necessary to develop a technological platform for the reliable and reproducible assessment of the probability of cancer initiation after transplantation of stem cell derivatives that were cultured in vitro and underwent various manipulations. Undoubtedly, the use of pluripotent stem cell lines requires regular monitoring of genetic and epigenetic integrity and testing of malignant tumorigenicity using adequate animal models.

To solve the problem of residual undifferentiated cells during in vitro differentiation of pluripotent stem cells, several strategies have been proposed to eliminate undifferentiated cells via genetic modification by using “suicide” gene expression [41–44] and the cytostatic exposure [45–48] to activate the proliferation arrest and cell death, as in cancer cells. However, another promising approach aims to correct the imbalance between proliferative and differentiation processes by enhancing differentiation with different combinations of differentiation inducers; this approach promotes the transformation of malignant teratocarcinomas toward benign mature teratomas [30, 49–51]. For instance, the well-known small molecular inducer of differentiation all-trans-retinoic acid is used for the clinical treatment of acute promyelocytic leukemia, since it intensifies the differentiation of undifferentiated tumor cells [49, 51]. Considering the substantial similarity of pluripotent stem and EC cells, a comparative analysis of the mechanisms that underlie their tumorigenic and carcinogenic potentials can allow us to find the most effective way of eliminating residual undifferentiated cells.

In the present study, we conducted a comparative analysis of the dynamics of in vitro and in vivo differentiation of the mouse ES and EC cells to identify differences in the mechanisms of differentiation and tumorigenic potentials of normal pluripotent stem cells and their malignant counterparts. Based on the obtained results, we attempted to identify ways to eliminate residual undifferentiated cells that are capable of initiating tumors after transplantation into immunodeficient recipient mice.

## 2. Materials and Methods

### 2.1. Cell Line Maintenance

Mouse ES R1 cell line was kindly provided by A. Nagy (Mount Sinai Hospital, Toronto, Canada), and mouse EC F9 cell line was obtained from the Russian Cell Culture Collection (http://www.rccc.cytspb.rssi.ru/). Mouse ES R1 cells were maintained on a mouse embryonic fibroblast feeder inactivated by mitomycin C (Sigma) or in a feeder-free system in a medium containing 10 ng/ml of leukemia inhibitory factor (Sigma). The ES R1 and EC F9 cells were cultivated in DMEM supplemented with 2 mM L-glutamine, 1% nonessential amino acids (HyClone), 0.1 mM *β*-mercaptoethanol (Sigma), and 15% Characterized Fetal Bovine Serum (HyClone) or 15% knockout serum replacement (Invitrogen).

### 2.2. Induction of ES and EC Cell Differentiation with Retinoic Acid and Exposure to Activin A, BMP4, PD98059, and LY294002

The differentiation of ES R1 and EC F9 cells was stimulated with retinoic acid (10^−6^ M, all-trans-retinoic acid, RA, Sigma). At the beginning of the experiment, the undifferentiated cells were plated in a density of 10,000 cells/cm^2^ and cultured overnight to facilitate adherence in a medium containing 1 mM L-glutamine, 1% nonessential amino acids, and 15% Characterized Fetal Bovine Serum (HyClone). The next day, the medium with serum was replaced with a medium supplemented with 15% knockout serum replacement and 10^−6^ M RA. Medium changes were conducted daily during experiments. After 5 days of RA stimulation, the ES R1 and EC F9 cells were dissociated using a 0.05% trypsin-EDTA solution (HyClone), transferred into new culture plates or flasks at a ratio of 1:3 and exposed to RA for the next 5 days (RA10). After 10 days of RA exposure, the cells were cultured in a standard medium without retinoic acid for 3 days (RA10 + 3).

In the next series of experiments to improve ES R1 cell differentiation, human Activin A (100 ng/ml, Invitrogen), human BMP4 (100 ng/ml, Sigma), PD98059 (50 *μ*M, Sigma), and LY294002 (25 *μ*M, Sigma) were added together with RA from day 5 to day 10. The differentiating ES R1 cells were then cultured in a medium without RA for 3 days.

### 2.3. Preparation of Cells for Transplantation

Before subcutaneous transplantation, differentiated ES R1 and EC F9 cells were dissociated using a 0.05% trypsin-EDTA solution (HyClone), and 10^6^ graft cells were concentrated in 50–70 *μ*l of Hanks solution (HyClone). For intraperitoneal transplantations, 5 × 10^5^ cells on day 10 of RA stimulation were plated onto acetate-cellulose membranes (CA-membrane, 1.2 cm^2^) and cultured in an 8-well Lab-Tek II Chamber Slide System (Nalge Nunc International) in a standard culture medium for 3 days.

### 2.4. Flow Cytometric Analysis of the Cell Cycle Distribution and Oct4-Expressing Cells

The cellular probes were analyzed using a Cytomics FC500 flow cytometer (Beckman Coulter). The cell suspensions (10^6^/ml) were prepared using the 0.05% trypsin-EDTA (HyClone) treatment. To analyze the cell cycle distribution, the cells were fixed with cold 70% ethanol. After fixation and triple washing with PBS the cells were incubated in PBS containing 20 *μ*g/ml of propidium iodide (Invitrogen/Molecular Probes) and 200 *μ*g/ml of RNAse A (Fermentas) for 30 min. After staining, the probes were analyzed immediately. The histograms were analyzed using the MultiCycle AV Software (Phoenix Flow Systems, USA).

For the flow cytometry analysis of Oct4-expressing cells, the suspensions of cells (10^6^/ml) were fixed with 3% paraformaldehyde in PBS for 15 min, washed with PBS, and treated with 0.5% Triton X-100, 3% bovine serum albumin, Fraction V (Sigma), and rabbit anti-Oct4 antibodies (1 : 200, Santa Cruz Biotechnology) in PBS for 40 min. After washing, the cells were incubated in a PBS solution containing 0.5% Triton X-100, 3% bovine serum albumin, and secondary chicken anti-rabbit antibodies conjugated with Alexa-488 (1 : 1000, Molecular Probes) for 30 min. For the negative control, the cells were treated with normal rabbit IgG (sc-3888, Santa Cruz Biotechnology) and then with the same secondary antibody solution described above.

### 2.5. Detection of Alkaline Phosphatase Activity (ALP) and Immunostaining

The ES R1 and EC F9 cells were fixed with 2% paraformaldehyde in phosphate-buffered saline, PBS, pH 7.0, within 15 min. ALP activity was detected after incubation in a solution containing 10 ml 0.02 M Tris-HCl buffer (pH 8.7), 1 mg Naphtol-AS-B1-phospate, and 5 mg fast red dye Texas Red (all from Sigma) at 37°C for 1 h.

For immunofluorescence analysis, cells fixed in 4% paraformaldehyde in PBS for 1 h were washed and permeabilized with 0.5% Triton X-100. Nonspecific reactions were blocked by 10% chicken serum (Gibco/Invitrogen). Primary rabbit anti-Oct4 and goat anti-Gata4 antibodies (Santa Cruz Biotechnology) were used at a dilution of 1 : 100. The cells were incubated in a solution of primary antibodies in PBS-Tween 20 at 4°C overnight. Secondary chicken anti-rabbit and donkey anti-goat antibodies conjugated with Alexa Fluor 594 and Alexa Fluor 488 (Molecular Probes) were diluted at 1 : 800 in a blocking buffer and applied to the cells for 4 h at room temperature. DAPI (Molecular Probes) was applied for 15 min for nuclear staining. The cells were mounted and examined under a Leica DMRXA2 fluorescence microscope (Leica Microsystems GmbH). For negative controls, the primary antibodies were omitted, and the same staining procedure was used.

### 2.6. RNA Isolation and Quantitative Real-Time PCR Analysis (qRT-PCR)

Total RNAs were extracted from mouse ESC and ECC samples using the TRIzol® Reagent (Invitrogen). The samples were treated with TURBO DNase (Ambion/Invitrogen) according to the manufacturer's recommendations. The RNA yield and quality were analyzed using the NanoDrop 2000 system (Thermo Scientific). cDNAs were synthesized using 1–1.5 *μ*g of total RNA, oligo-dT18 primer, and Maxima Reverse Transcriptase (Fermentas) according to the manufacturer's protocols.

A relative quantitative analysis of gene expression was carried out using the Applied Biosystems Real-Time PCR System 7500 (Life Technologies). The probes were prepared using the qRT-PCR master mix with SYBR Green and ROX passive reference dye (Evrogen, Russia). The following amplification protocols were used: denaturation at 95°C for 5 min, followed by 40 cycles at 95°C for 15 sec and at 60°C for 1 min. All experiments were run in triplicate. The expression levels of target mRNAs were normalized to the expression of the housekeeping gene hypoxanthine guanine phosphoribosyltransferase (Hprt). The relative levels of target gene expression were calculated using the comparative 2^−∆∆Ct^ method (ABI Relative Quantification Study software, Applied Biosystems).

Specific primers were designed using GenBank and Ensemble data for the annotated sequences of the target genes using the Beacon Designer 8.0 software (PREMIER Biosoft, USA) and Primer 3. The primer sequences and sizes of their expected products are represented in Table S1 (Supplemental information available online at https://doi.org/10.1155/2017/7284872). The gene expression data were subjected to statistical analysis using the R v.3.2.3 software (http://www.r-project.org). The averages of the gene expression data obtained from three independent experiments were used for statistical analysis using one-way ANOVA followed by a Tukey's post hoc test.

### 2.7. Teratoma and Teratocarcinoma Assay

To study the development of teratomas and teratocarcinomas, 10–12-week-old immunodeficient nude mice (Nu/Nu) delivered from the Animal Breeding Facility-Branch “Pushchino” of Shemyakin and Ovchinnikov Institute of Bioorganic Chemistry, Russian Academy of Sciences (stock line was obtained from the Charles River Laboratories Inc., Wilmington, MA) were used as recipients. Nude mice were kept under pathogen-free conditions. The animal keeping and all experiments were approved by the Ethics Committee of the Institute of Developmental Biology, Russian Academy of Sciences, and performed in accordance with the Russian Federation legislation (Order of the Ministry of Health and Social Development of the Russian Federation Number 708n, August 28, 2010) based on the European Convention for the Protection of Vertebrate Animals used for Experimental and Other Scientific Purposes. Before the subcutaneous and intraperitoneal cell transplantation procedures, the animals were anesthetized with an intraperitoneal injection of 100 mg/kg ketamine (MEZ, Russia) and 10 mg/kg xylazine (Rometar, Spofa, Czech Republic). In subcutaneous transplantation experiments, differentiating ES R1 and EC F9 cells (0.5–1 × 10^6^ cells per mouse) were injected under the skin of the neck area of nude mice using 1 ml syringes with 27G needles (Becton Dickinson). During intraperitoneal cell transplantation, midline incisions were made in the skin and body wall to get access to the peritoneal cavity. The cells on the CA-membrane were transferred into the peritoneal cavity using tweezers.

At the end of the experiments (6–30 weeks after transplantation), the animals were sacrificed by cervical dislocation or by injection of a lethal dose of intravenous barbiturates. Autopsies were performed for all mice. The developed teratomas and teratocarcinomas were isolated and fixed with 10% paraformaldehyde (Sigma), dehydrated according to the standard method, and embedded in paraffin for sectioning. Histological preparations were stained with hematoxylin and eosin and examined under a Leica DM RXA2 microscope.

## 3. Results

### 3.1. Dynamics of RA-Induced Differentiation of ES and EC Cells

**Figure 1:**
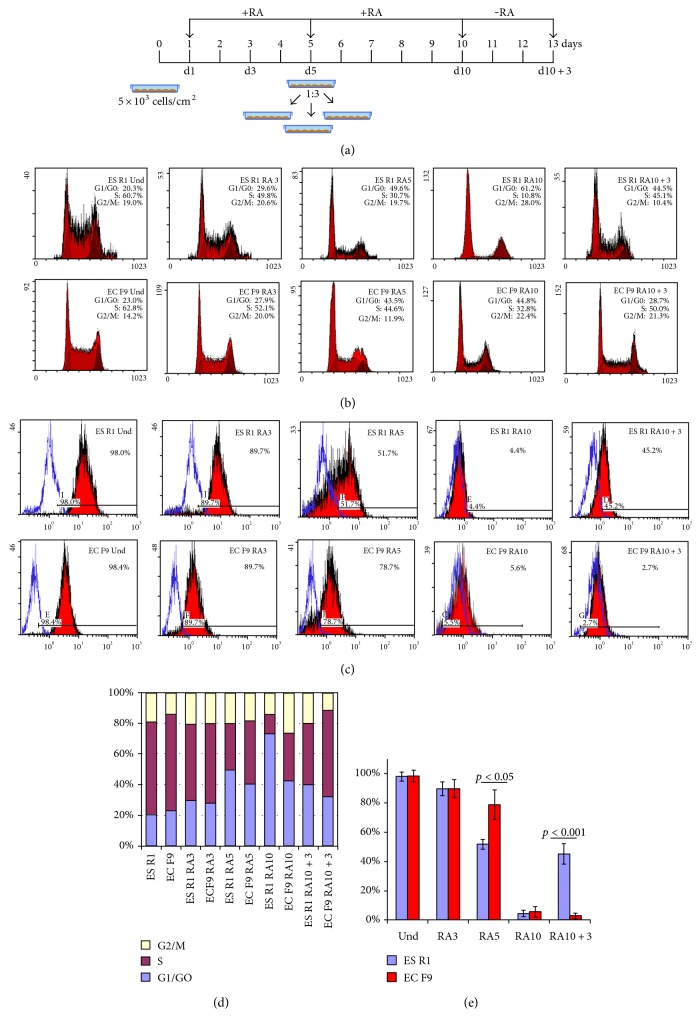
Proliferative activity and differentiation dynamics of ES R1 and EC F9 cells after 10 days of RA stimulation and 3 days after RA withdrawal. (a) Experimental design of RA-induced differentiation of ES R1 and EC F9 cells. (b and d) Flow cytometric analysis of the distributions of the cell cycle stages in the populations of RA-stimulated ES R1 and EC F9 cells. (c and e) Flow cytometric analysis of the number of Oct4-expressing cells in the populations of RA-stimulated ES R1 and EC F9 cells. The data are represented as the mean ± s.e., ANOVA.

**Figure 2:**
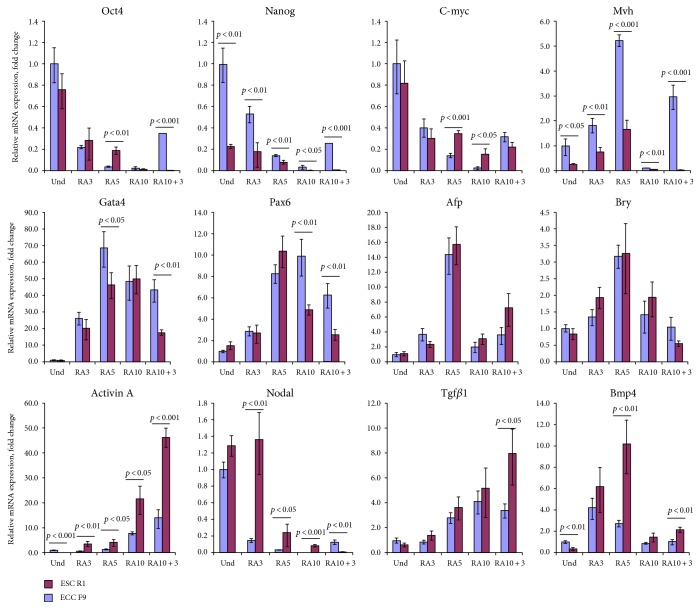
The gene expression patterns of pluripotency and embryonic lineage markers and TGF*β* factors in the course of RA-induced differentiation of ES R1 and EC F9 cells. The gene expression levels of each gene in differentiated cells were evaluated relative to the gene expression levels in undifferentiated ES R1 cells. The data are represented as the mean ± s.d.; significant differences were estimated using ANOVA.

To clarify the mechanisms that regulate the balance of proliferation and differentiation processes in normal pluripotent and malignant teratocarcinoma cells, the proliferative activity and dynamics of in vitro differentiation of ES R1 and EC F9 cells were analyzed after RA stimulation (Figure 1(a)). During 10 days of RA-induced differentiation, there is a gradual decrease in the number of cells in the S-phase of the cell cycle (from 60% to 10–30%) and an increase of the number of cells in the G1/G0-phase (from 10–20% to 45–60%) in both the ES R1 and EC F9 cell populations (Figures 1(b) and 1(d)). However, the number of cells in the S-phase of the cell cycle is significantly lower in the populations of differentiating ES R1 cells than in the EC F9 cell populations on day 5 (30.7% versus 44.6%, resp.) and day 10 (10.8% versus 32.8%, resp.) of RA exposure (Figures 1(b) and 1(d)). On day 3 after RA withdrawal (RA10 + 3), the number of cells in the in the S-phase of the cell cycle is similar in both cell lines, while the number of cell in the G1/G0-phase is significantly higher in differentiating ES R1 cells (Figures 1(b) and 1(d)). Similarly, lower expression levels of C-myc are detected in the ES R1 cells than in the EC F9 cells on days 5 and 10 of RA-induced differentiation (Figure 2).

**Figure 3:**
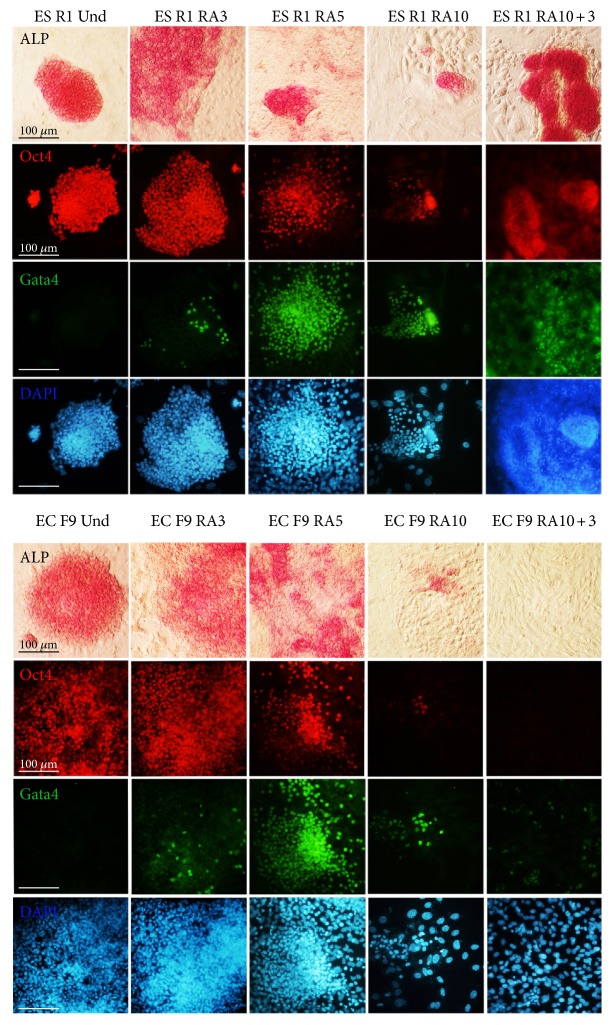
ALP activity and expression of Oct4 and Gata4 in differentiating ES R1 and EC F9 cells after RA stimulation for 10 days and 3 days after RA withdrawal. Nuclei were counterstained with DAPI. A significant number of the ES R1 and EC F9 cells expressed both Gata4 and Oct4 at all stages of RA-stimulated differentiation. Scale bar = 100 *μ*m.

An analysis of differentiation dynamics identified a gradual decrease in the number of Oct4- and ALP-expressing cells in the ES R1 and EC F9 cell populations (Figures 1(c) and 1(e), Figure 3). However, the numbers of Oct4-expressing cells differ between the ES R1 and EC F9 cell populations on day 5 of RA-induced differentiation (52% versus 78.8%, resp.) and on day 3 after RA withdrawal (45.2% versus 2.7%, resp.). No significant differences are detected on day 10 (Figures 1(c) and 1(e)). These dynamics were confirmed by qRT-PCR analysis of Oct4 expression in differentiating ES R1 and EC F9 populations (Figure 2). Thus, during the early stages of RA stimulation, proliferation and differentiation dynamics of ES R1 and EC F9 cells are similar, but from the 5th to the 10th day of the experiment, there are significant differences in the studied cellular characteristics, which become most pronounced on day 3 after RA withdrawal.

### 3.2. Gene Expression Analysis of Embryonic Lineage Commitment in the Course of RA-Induced Differentiation of ES and EC Cells

To investigate early embryonic lineages during RA-induced differentiation of ES R1 and EC F9 cells, gene expression of pluripotency (Oct4 and Nanog) and lineage markers (Mvh, Gata4, Pax6, Afp, and Bry) was studied (Figures 2 and 3). Moreover, endogenous expression of TGF*β* family factors (TGF*β*1, Activin A, Nodal, and BMP4), which play a key role in the early lineage differentiation, was also examined (Figure 2). There is higher expression of Nanog and Mvh (germ line marker) in the ES R1 cell populations than in the EC F9 cells at all stages of RA-induced differentiation. Higher expression of Oct4 is detected in the EC F9 cells on day 5 of RA stimulation and in the ES R1 cells on day 3 after RA withdrawal (Figure 2). The expression of most somatic cell lineage markers increases significantly during the differentiation of the ES R1 and EC F9 cells (Figure 2). The strongest increase in expression is observed for markers of extraembryonic endoderm (Gata4) and neuroectoderm (Pax6) in differentiating ES R1 and EC F9 cells. Gata4 expression significantly differs between the ES R1 and EC F9 cells on day 5 of RA stimulation and on day 3 after RA withdrawal, while Pax6 expression differs between ES R1 and EC F9 cells during late stages of differentiation (RA10 and RA10 + 3). However, no significant differences in the expression of endoderm and mesoderm markers (Afp and Bry, resp.) are found between differentiating ES R1 and EC F9 populations (Figure 2).

In undifferentiated and differentiated ES R1 and EC F9 cells, endogenous expression of TGF*β* family factors that initiate the corresponding signaling pathways also differs significantly. The most substantial differences are revealed in patterns of endogenous expression for Activin A and Nodal. In differentiating ES R1 and EC F9 cell populations, the expression of Activin A is upregulated, while the expression of Nodal is downregulated. Activin A is expressed at high levels in undifferentiated ES R1 cells, but the expression of this factor increases sharply and becomes higher in EC F9 cells during all stages of RA-induced differentiation (Figure 2). In contrast, Nodal is expressed at similar levels in undifferentiated ES R1 and EC F9 cells but decreases more strongly during differentiation of the ES R1 cells (Figure 2). Endogenous expression of TGF*β*1 gradually increases during differentiation in both cell lines but differs significantly between ES R1 and EC F9 cells only during the final stage (RA10 + 3).

In contrast, the expression pattern of BMP4 differs from the Activin A and Nodal patterns during the differentiation of ES R1 and EC F9 cells. Endogenous expression of BMP4 is upregulated on days 3 and 5 and downregulated during the late stages of differentiation in both studied cell lines (Figure 2). These data show that differences in the endogenous expression of Activin, Nodal, and BMP that activate corresponding signaling pathways can contribute to differences in the dynamics of RA-induced lineage differentiation of ES R1 and EC F9 cells.

### 3.3. Tumorigenic Potential of RA-Simulated ES and EC Cells after Transplantation into Nude Mice

**Figure 4:**
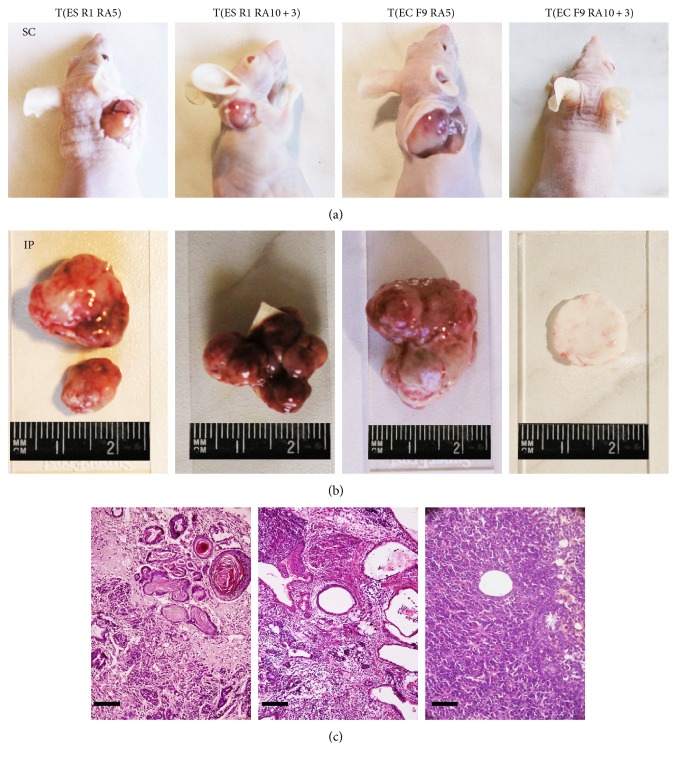
Tumorigenic and differentiation potential of RA-simulated ES R1 and EC F9 cells after transplantation into nude mice. (a and b) teratomas and teratocarcinomas developed in nude mice after subcutaneous (SC) and intraperitoneal (IP) transplantation of ES R1 and EC F9 cells on day 5 RA stimulation (T(ES R1 RA5), T(EC F9 RA5)) and on day 3 after RA withdrawal (T(ES R1 RA10 + 3), T(EC F9 RA10 + 3)). (c) Sections through teratomas and teratocarcinomas formed by RA-simulated ES R1 and EC F9 cells after transplantation into nude mice. The teratomas contain derivatives of three germ layers while the teratocarcinomas consisted entirely of undifferentiated cancer cells. No teratocarcinomas have formed during 30 weeks after the transplantation of EC F9 cells on day 3 after RA withdrawal (RA10 + 3). Scale bar = 100 *μ*m.

To evaluate tumorigenic potential, differentiating ES R1 and EC F9 cells were transplanted subcutaneously or intraperitoneally into nude mice. According to our previous data on the dynamics of tumor development [21], the formation of teratomas and teratocarcinomas was monitored in two tissue transplantation sites for 6–30 weeks (Table 1). Within 5-6 weeks, teratomas and teratocarcinomas develop in all nude mice (100%) after the transplantation of ES R1 and EC F9 cells on day 5 of RA stimulation in vitro (Figure 4, Table 1). Only teratomas develop in all mice after the transplantation of ES R1 cells on day 3 after RA withdrawal (RA10 + 3), and no teratocarcinomas form within 30 weeks after the transplantation of EC F9 cells at the same stage of in vitro differentiation (Figure 4, Table 1). The formation of tumors outside the transplantation sites is not observed after the transplantations of the studied cells. Teratomas that develop after the transplantation of differentiating ES R1 cells contain different cell derivatives of three germ layers (Figure 4(c)). In contrast, the teratocarcinomas formed by the EC F9 cells on day 5 of in vitro RA stimulation (RA5) consist of entirely undifferentiated teratocarcinoma cells (Figure 4(c)). These experiments demonstrate different tumorigenic and differentiation potentials of the ES R1 and EC F9 cell populations after RA stimulation in vitro.

### 3.4. Effects of Stimulation of the Activin A/Nodal and BMP Signaling Pathways and Inhibition of the MEK/ERK and PI3K/Act Signaling Pathways on RA-Induced Differentiation of ES R1 Cells

**Figure 5:**
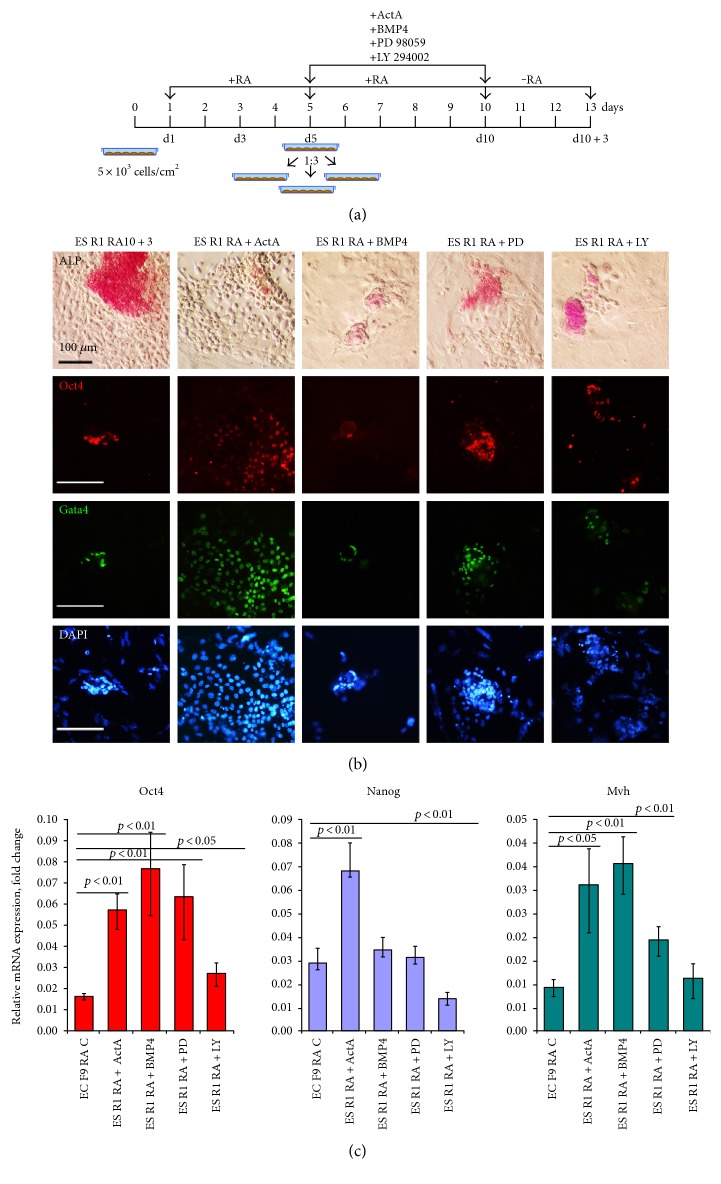
Stimulation of the Activin A/Nodal and BMP signaling pathways and inhibition of the MEK/ERK and PI3K/Act signaling pathways in ES R1 cells during the course of RA-induced differentiation. (a) Design of experiments on the induced differentiation of ES R1 cells with RA and exposure to Activin A, BMP4, PD98059, and LY294002. (b) ALP activity and Oct4 and Gata4 expression in differentiating ES R1 cells after exposure to RA, Activin A, BMP4, PD98059, and LY294002 on day 3 after RA and additive withdrawal. Nuclei were counterstained with DAPI. ActA: Activin A; BMP: BMP4; PD: PD98059; LY: LY294002. Scale bar = 100 *μ*m. (c) The expression of Oct4, Nanog, and Mvh in differentiating ES R1 after exposure to RA, Activin A, BMP4, PD98059, and LY294002 on day 3 after RA and additive withdrawal. The gene expression levels of each gene in all experimental variants of differentiating ES R1 cells were calculated relative to the gene expression levels in control ES R1 cells on day 3 after RA withdrawal (ES R1 RA10 + 3) and statistically evaluated relative to the gene expression levels in control EC F9 cells on day 3 after RA withdrawal (EC F9 RA10 + 3). The data are represented as the mean ± s.d.; significant differences were estimated using ANOVA.

Based on our findings concerning the differences in in vitro and in vivo differentiation of the ES R1 and EC F9 cells, we hypothesized that the tumorigenic potential of ES R1 and EC F9 cells on day 10 after RA stimulation could be associated with different activity levels of TGF*β* family signaling pathways, as well as the MEK/ERK and PI3K/Act signaling cascades that counterbalance TGF*β* family signaling pathways. The effects of modulation of these signaling pathways on the differentiation and tumor potential via enhancing the activity of Activin A and BMP4 signaling pathways and inhibiting of MEK/ERK and PI3K/Act signaling pathways in RA-stimulated ES R1 cells were studied in the next series of experiments. The ES R1 cells were exposed to factors (Activin A and BMP) and inhibitors (PD98059 and LY294002) from day 5 to day 10 of RA stimulation cells, because the most significant differences between differentiating ES R1 and ECF9 cells are detected during these stages. The design of the experiment is shown in Figure 5(a).

**Figure 6:**
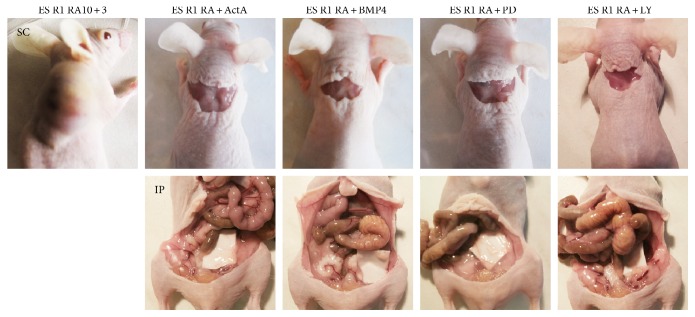
Assessment of the tumorigenic potential of ES R1 cells stimulated to differentiate with RA, Activin A, BMP4, PD98059, and LY294002 after transplantation into nude mice. No tumors were detected in nude mice after subcutaneous (SC) and intraperitoneal (IP) transplantation of ES R1 cells on day 3 after RA and additive withdrawal. Teratomas formed only after subcutaneous transplantation of ES R1 cells on day 3 after RA withdrawal (ES R1 RA10 + 3). ActA: Activin A; BMP: BMP4; PD: PD98059; LY: LY294002.

In all experimental variants, small numbers of Oct4- and ALP-positive cells are identified at the final stage of experiments. The numbers are similar to that observed on day 10 of RA stimulation alone (Figure 3 and Figure 5(b)). Moreover, the expression levels of Oct4, Nanog, and Mvh are significantly lower in differentiating ES R1 cell populations exposed to RA and additives than that in controls (ES R1 RA10 + 3) and are comparable to the expression levels in differentiating EC F9 cells (EC F9 RA10 + 3) (Figure 5(c)). An analysis of the tumorigenic potential of ES R1 cells differentiated with exposure to RA and additives shows that no tumors develop in either tissue site of nude mice 30 weeks after transplantation of all differentiating ES cell populations (Table 2 and Figure 6).

## 4. Discussion

Our study of the differentiation dynamics of normal pluripotent stem and malignant teratocarcinoma cells was conducted to answer the following questions: (i) Why do residual undifferentiated cells remain after induced in vitro differentiation of ES and EC cells? (ii) Are the residual undifferentiated cells tumorigenic and carcinogenic? (iii) What possible mechanisms underlie incomplete differentiation of ES and EC cells? And (iv) how can we reduce the number of residual undifferentiated cells in differentiating ES cell populations that are capable of forming tumors after transplantation into recipients?

The present study revealed significant differences in proliferative activity and differentiation dynamics between the ES R1 and EC F9 cells from day 5 to day 10 of RA-induced differentiation. However, despite similar numbers of residual Oct4-expressing cells in both ES and EC cell populations on day 10, the tumorigenic potential of these cells is strikingly different. Unexpectedly, differentiating ES R1 cell populations retain the ability to form tumors (teratomas), whereas the EC F9 cell populations lose tumorigenicity, as reported previously [30]. These findings indicate a different status of residual Oct4-expressing cells in differentiating ES and EC cell populations.

**Figure 7:**
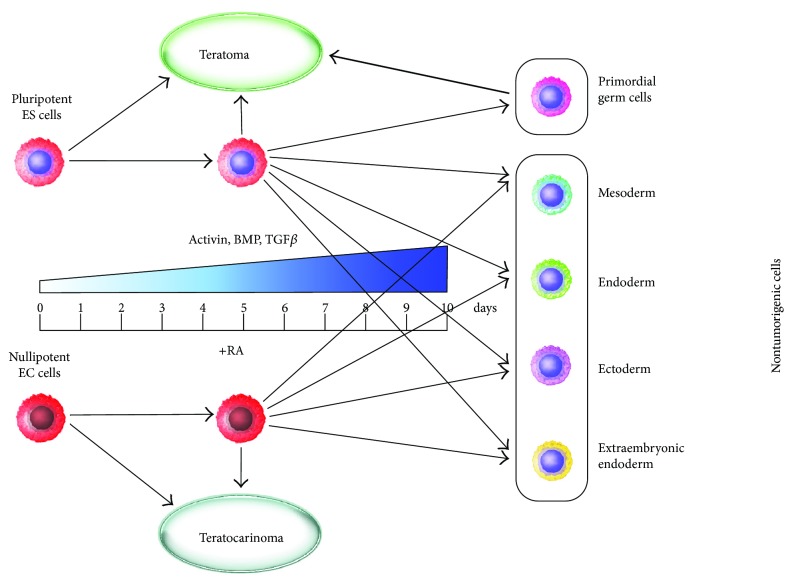
Tumorigenic and differentiation potentials of ES R1 and EC F9 cells during the course of RA-induced differentiation.

A gene expression analysis of pluripotent and lineage markers reveals significantly higher expression levels of Nanog and Mvh in ES R1 cells than in EC F9 cells at all stages of differentiation indicating a possible role of these genes in the maintenance of differentiation and tumorigenic potentials. Previously, we suggested that Mvh, which is a regulator of embryonic and adult germ cell development, may play a role in maintaining of the naive pluripotent state of mouse and human ES cells [52–56]. In this context, the residual undifferentiated ES cells with high expression of Oct4, Nanog, and Mvh can be considered to be the earliest precursors of germ cells, similar to primordial germ cells before their colonization of the gonadal ridges. This assumption is supported by data that demonstrate the easy in vitro conversion of primordial germ cells into EG cells, which are very similar to ES cells [55, 57–60]. Consequently, higher expression of Mvh may indicate a stronger ability of the 10-day RA-stimulated ES R1 cells to differentiate into primordial germ cells, which also easily form teratomas after transplantation into nude mice. Therefore, we propose that the ability of pluripotent stem cells to differentiate into primordial germ cells is associated with their ability to develop teratomas containing all types of embryonic cell derivatives. The residual undifferentiated ES cells cannot be considered to be cancerous because unlike EC cells, these cells are capable of differentiation into precursors of three germ layers in teratomas and even in secondary teratomas after recloning and secondary transplantation [21]. Presumably, the residual undifferentiated ES cells represent an intermediate cell type between pluripotent and primordial germ cells which are both able to develop teratomas. In contrast, EC F9 cells expressing low levels of Mvh can differentiate after RA-stimulation only in the somatic nontumorigenic derivatives (Figure 7).

The mechanisms of RA-induced differentiation of EC and ES cells have been studied extensively [30, 49, 61–67]. To investigate the possible mechanisms underlying various tumor and differentiation potentials of ES R1 and EC F9 cells, we focused on TGF*β* family factors that play a key role in the regulation of lineage fate during the early development and the earliest stages of pluripotent stem cell differentiation [68–70]. The observation that the expression levels of Activin A, Nodal, and BMP4 were higher in the EC F9 cells than in the ES R1 cells, particularly between the 5th and 10th day of RA-stimulation, led us to propose that additional stimulation of these signaling pathways might enhance the differentiation of the ES R1cells. Indeed, modulation of the Activin/Nodal and BMP signaling cascades via exposure to exogenous Activin A and BMP4 factors or by inhibiting the MEK/ERK and PI3K/Act-signaling pathways that counterbalance canonical TGF*β* family factor signaling pathways resulted in reduced expression of Mvh in all experimental cell populations and a loss of tumorigenicity after transplantation into nude mice. Thus, these data show that the differentiation strategy of modulating prodifferentiation and antiproliferative signals by stimulating the Activin A/Nodal or BMP signaling pathways or inhibiting the MEK/ERK and PI3K/Act signaling pathways during the time window from 5 to 10 days of ES cell differentiation may be effective for significantly reducing of the number of cells that initiate teratoma development (Figure 7). Interestingly, this strategy is also effective for EC F9 cells, which express higher endogenous levels of Activin A and BMP4 in response to RA stimulation.

Most of the developed protocols for the in vitro differentiation of pluripotent stem cells are based on the knowledge of the molecular mechanisms that regulate the commitment and differentiation of early embryonic and extraembryonic cells during development. The modulation of the MEK/ERK, PI3K/Act, Wnt, and TGF*β* family signaling pathways using various cocktails of growth factors and inhibitors, as well as small molecular inducers, for a different time of exposure is an effective approach for deriving the required differentiated cell types [68–77]. In most cases, the cell populations differentiated in vitro are heterogeneous and require cell sorting. However, the residual undifferentiated cells are a minor subpopulation; therefore, to remove these cells, negative selection methods are used that involve activating cell death in target cells via a “suicide” gene approach and chemical inducers [41–48, 78]. Nevertheless, the application of these technologies may have negative effects on the viability of the desired differentiated cells and may cause uncontrolled genetic rearrangements due to insertional mutagenesis [43–45, 79]. Therefore, the strategy of modulating signaling pathways to enhance differentiation may be safer and more effective. Our study demonstrates the effectiveness of this strategy for inhibiting the tumorigenicity of residual undifferentiated ES cells. However, this strategy requires additive efforts to create the most effective protocols for increasing the effectiveness of directed differentiation and reducing the number of residual undifferentiated cells.

## 5. Conclusions

The present comparative study of RA-induced differentiation the ES R1 and EC F9 cells aimed to clarify the possible mechanisms underlying the incomplete in vitro differentiation of these cells and to determine a new approach eliminating residual undifferentiated ES cells, which can form tumors after transplantation into recipients. During 10 days of RA-induced differentiation, ES R1 and EC F9 cells were found to exhibit differences in proliferative activity and differentiation dynamics in vitro, as well as different tumorigenic potential after transplantation into nude mice. Importantly, differentiating ES R1 cell populations retain the ability to form teratomas, while the EC F9 cell populations lose tumorigenicity. Gene expression analysis revealed higher expression of Nanog and Mvh, as well as Activin A and BMP4, in undifferentiated ES R1 cells in comparison to EC F9 cells. However, the expression levels of Activin A and BMP4 increase more sharply in the EC F9 cells after RA stimulation. Moreover, the stimulation of the Activin/Nodal and BMP signaling cascades after exposure to exogenous Activin A and BMP4 factors and inhibitors of the MEK/ERK and PI3K/Act signaling pathways results in a reduction of the number of residual Oct4-expressing ES R1 cells and a loss of tumorigenicity. Thus, our study demonstrates that a differentiation strategy that enhances the Activin A/Nodal and BMP signaling pathways or inhibits the MEK/ERK and PI3K/Act signaling pathways in ES cells may be effective for reducing the number of tumorigenic cells. This approach may promote progress in the development of safe stem cell therapeutics.

## Supplementary Material

Table S1. Real–time reverse transcription polymerase chain reaction (qRT–PCR) primers

## Figures and Tables

**Table 1:** Tumor development after transplantations of RA-stimulated ES R1 and EC F9 into immunodeficient nude mice.

Transplantation sites	ES R1 + RA5	ES R1 + RA10	EC F9 + RA5	EC F9 + RA10
SC	3/3 (100%), 6 wk	4/4 (100%), 6 wk	3/3 (100%), 6 wk	0/6 (0%), 30 wk
IP	3/3 (100%), 6 wk	4/4 (100%), 6 wk	3/3 (100%), 6 wk	0/8 (0%), 30 wk

The percentage and number of animals are indicated in which tumors were found in the transplantation sites during autopsy. SC: subcutaneous transplantation; IP: intraperitoneal transplantation (the cells grown on acetate cellulose membranes).

**Table 2:** Tumor development in nude mice after transplantations of ES R1 stimulated with RA in combination with Activin A, BMP4, PD98059, and LY294002.

Transplantation sites	ES R1 + RA + ActA	ES R1 + RA + BMP4	ES R1 + RA + LY	ES R1 + RA + PD
SC	0/5 (0%), 30 wk	0/5 (0%), 30 wk	0/5 (0%), 30 wk	0/5 (0%), 30 wk
IP	0/5 (0%), 30 wk	0/5 (0%), 30 wk	0/5 (0%), 30 wk	0/5 (0%), 30 wk

The percentage and number of animals are indicated in which tumors were found in the transplantation sites during autopsy. SC: subcutaneous transplantation; IP: intraperitoneal transplantation (the cells grown on acetate cellulose membranes).
