# Supplementary material for: Tumorigenic and Differentiation Potentials of Embryonic Stem Cells Depend on TGFβ Family Signaling: Lessons from Teratocarcinoma Cells Stimulated to Differentiate with Retinoic Acid
Source: Stem Cells Int. 2017 Jul 16;2017:7284872. doi: 10.1155/2017/7284872 (PMC5534322; doi:10.1155/2017/7284872)
Supplement: Supplementary file 1 — Table S1. Real–time reverse transcription polymerase chain reaction (qRT–PCR) primers [file 7284872.f1.doc]

Table S1. Real-time reverse transcription polymerase chain reaction (qRT-PCR) primers

| Gene | Accession no. | Primer sequences | Amplicon (bp) |
| --- | --- | --- | --- |
|  |  |  |  |
| *Hprt* | NM_013556.2 | 5' ttgggcttacctcactgctttc 3'  5' ctaatcacgacgctgggactg 3' | 125 |
| *Oct4* | NM_013633.2 | 5' caccctgggcgttctctttg 3'  5' gttctcattgttgtcggcttcc 3' | 142 |
| *Nanog* | NM_028016.3 | 5' aactctcctccattctgaacctga 3'  5' ggtgctgagcccttctgaatc 3' | 136 |
| *Mvh* | NM_010029.2  NM_001145885.1 | 5' aggaatgccatcaaaggaacaac 3'  5' gcccaacagcgacaaacaag 3' | 119 |
| *Gata4* | NM_008092 NM_001310610.1 | 5' tctcactatgggcacagcag 3'  5' gggacagcttcagagcagac 3' | 100 |
| *Afp* | NM_007423.4 | 5' cttccctcatcctcctgctac 3'  5' acattcttctccgtcacgca 3' | 109 |
| *Pax6* | NM_013627.6 | 5' taccagtgtctaccagccaatcc 3'  5' gcacgagtatgaggaggtctga 3' | 193 |
| *Bry* | NM_009309.2 | 5' atgctgcctgtgagtcataac 3'  5' cgtg tgc gtc agt ggt gtg 3' | 177 |
| *C-myc* | NM_001177352 NM_010849.4 | 5' tgatgtggtgtctgtggagaaga 3'  5' gcgtagttgtgctggtgagt 3' | 144 |
| *ActivinA/* *Inhba* | NM_008380.2 | 5' tggagcagacctcggagatcatcac 3'  5' ttggtcctggttctgttagccttgg 3' | 160 |
| *Nodal* | NM_013611.5 | 5' gcgagtgtcctaaccctgtg 3'  5' atgctcagtggcttggtc 3' | 136 |
| *Tgfb1* | NM_011577.2 | 5'caattcctggcgttaccttgg 3'  5'ccctgtattccgtctccttgg 3' | 120 |
| *Bmp4* | NM_007554.3 | 5'tctggtctccgtccctgatg3'  5'cgctccgaatggcactacg3' | 175 |
